# Prenatal exposure to alcohol: mechanisms of cerebral vascular damage and lifelong consequences

**DOI:** 10.3389/adar.2022.10818

**Published:** 2022-11-21

**Authors:** Partha S. Saha, William G. Mayhan

**Affiliations:** Division of Basic Biomedical Sciences, Sanford School of Medicine, University of South Dakota, Vermillion, SD, United States

**Keywords:** brain, cerebral resistance arterioles, prenatal alcohol exposure, FASD, cerebral blood flow

## Abstract

Alcohol is a well-known teratogen, and prenatal alcohol exposure (PAE) leads to a greater incidence of many cardiovascular-related pathologies. Alcohol negatively impacts vasculogenesis and angiogenesis in the developing fetal brain, resulting in fetal alcohol spectrum disorders (FASD). Ample preclinical evidence indicates that the normal reactivity of cerebral resistance arterioles, which regulate blood flow distribution in response to metabolic demand (neurovascular coupling), is impaired by PAE. This impairment of dilation of cerebral arteries may carry implications for the susceptibility of the brain to cerebral ischemic damage well into adulthood. The focus of this review is to consolidate findings from studies examining the influence of PAE on vascular development, give insights into relevant pathological mechanisms at the vascular level, evaluate the risks of ethanol-driven alterations of cerebrovascular reactivity, and revisit different preventive interventions that may have promise in reversing vascular changes in preclinical FASD models.

## Introduction

One of the most often used psychotropic substances, ethanol, permeates the placental barrier, and hence exerts potential teratogenic effects. A 2011 survey found that 45 percent of pregnancies in the United States were unintentional, with one in ten pregnant women between the ages of 18 and 44 reporting intake of alcohol in the previous 30 days and one in thirty-three reporting binge drinking [1, 2]. The pattern and intensity of the detrimental effects of prenatal alcohol exposure are dependent on alcohol dose, timing, sequence, and persistence of alcohol intake. All current research indicates that alcohol has a detrimental effect on fetal development. However, the fetal brain is the most substantially afflicted organ, displaying structural and functional abnormalities as a result of maternal alcohol consumption [3, 4]. Exposure to alcohol may result in fetal alcohol spectrum disorders (FASDs) in humans. FASD is a collective name for the harmful effects of prenatal alcohol exposure (PAE), including impairments in physiologic, neurologic, and behavioral development [5]. It should also be noted that FASD is an umbrella term that incorporates a variety of alcohol-related disorders with a range of severity. The most severe type of FASD, fetal alcohol syndrome (FAS) is characterized by specific physical traits in neonates. Partial FAS (pFAS), on the other hand, is diagnosed when there is validated prenatal alcohol exposure but not enough signs and symptoms to confirm a diagnosis of FAS [6]. Recent investigations, however, have seen the effects of alcohol on fetal cerebrovascular function emerge as a key mediator since brain energy needs are usually fulfilled by a continually adjusting blood supply [7]. Such an adaptation begins at the level of cerebral arteries and extends to microvessels that enter the brain parenchyma and form the neurovascular unit [8,9]. PAE may have acute and chronic teratogenic and toxic impacts on cerebrovascular physiology. This review’s objective is to outline the pathological pathways in different developmental phases, as well as their morphological and functional consequences on the cerebral circulation. It is held that alterations of cerebral blood flow (CBF) owing to dysregulation of cerebral blood vessels in PAE may be a significant contributor to the etiology of several cerebrovascular events, such as stroke.

## Cerebrovascular effects of PAE during fetal development

During the early-gestation period, the neural tube is surrounded by a perineural vascular plexus (PNPV) developed by vasculogenesis, the process of blood vessel development in the embryo, which involves the *de novo* generation of endothelial cells (ECs). Beginning in the mid-gestation period, endothelial cells from the PNPV penetrate the neural parenchyma, utilizing fibers from multipotent stem cells, like radial glia cells from the developing forebrain, to direct their migration toward the ventricular surface, thereby initiating the first vessels of the CNS. The second trimester is crucial for neuronal and blood vessel growth in the fetal brain [10, 11]. During this phase, a network of arteries inside the sub-arachnoid space gives birth to microvessels that enter the fetal brain [11]. This emerging vasculature supports nutritional requirements and endocrine regulation of fetal brain development [12]. In the early-mid gestation period, vascular development in the CNS is mediated by angiogenesis, which is defined as a series of cellular and molecular processes including the production of new vessels and culminates in the formation of the blood-brain barrier (BBB) [13,14,15]. At the mid-late gestation period, interactions among neural cells and ECs are initiated and continue through late-gestation and until birth. Communication between glial cells and the vasculature is crucial for the optimal development of the nervous system [16]. Pericytes increase vascular stability by releasing a variety of stabilization factors, such as angiopoietin-1 (ANG1) and platelet-derived growth factor (PDGF-β), tissue inhibitor of metalloproteinase 3 (TIMP3) [17]. In response to neural impulses at the perivascular terminal, astrocytes produce chemicals capable of regulating vascular tone. From the late-gestation period until birth, the BBB continues to mature *via* the expression of various molecules by the BBB’s cellular components, culminating in the development of a basal membrane rich in laminin, collagen IV, and fibronectin that fully surrounds the brain vasculature [18].

It has been established that maternal ethanol exposure causes rapid and persistent loss of blood flow from the umbilical artery to the fetal brain, potentially distressing nutrition and the maternal/fetal endocrine environment during a critical stage for neurogenesis and angiogenesis in the developing brain [19]. Because alcohol exposure during pregnancy is known to alter brain development, the majority of investigations have centered on neural cells. At the molecular and subcellular levels, however, cerebrovascular development is a more complicated, multi-step process involving a large number of molecular participants.

Angiogenesis is a complex process that is delicately controlled by a balance between pro-and anti-angiogenic factors [20]. They consist of, but are not limited to, vascular endothelial growth factor (VEGF), integrins, fibronectin, angiopoietins, vascular cell adhesion molecules (VCAM), fibroblast growth factors (FGF), tyrosine kinases (TK), extracellular matrix (ECM) proteins and proteinases, transforming growth factor (TGF), and Wnt signaling factors as growth-stimulatory signals. On the other hand, tumor necrosis factor (TNF) signaling and pro-apoptosis factors are stop signals [21, 22, 23]. Recent research conducted on adult nonhuman primates [24] and mice [25, 26] conclusively demonstrates that alcohol has a deleterious effect on vasculogenesis and angiogenesis pathways. According to microarray investigations, genes (e.g., VEGF gene family) implicated in angiogenesis are molecular targets of ethanol toxicity [27].

In addition to disrupting the signaling components that regulate vascularization and brain development, PAE may affect molecular targets that are particular to cerebrovascular development. Ethanol may affect the molecular and cellular elements of the BBB directly as soon as they are present during CNS development. Other research indicated that the embryonic brain is more susceptible to ethanol due to the high prevalence of proapoptotic proteins and low expression of proteins associated with stress response systems, namely autophagy or unfolded protein response [28]. Also, a proteomics investigation revealed that alcohol-exposed baboon fetuses mainly had changes in mitochondrial and structural proteins in their cerebral arteries. Unlike mitochondrial proteins, structural proteins were downregulated in the brain of fetal arteries exposed to alcohol [29].

## Morphological damage in cerebral blood vessels due to PAE

Considering ethical limits of human-based research, the PAE of laboratory animals has been extensively used as an alternative. Results from these animal models showed a significant influence on the generation and expression of microvessels in the rodent model. Besides dose, the timing of ethanol exposure has a significant impact on the outcome of fetal brain development. Since the gestational period of rodents (i.e., 18–23 days) is much shorter than that of humans, the morphological and functional effects of alcohol on these animals may need to be extrapolated to equivalent pregnancy terms in humans. In both rats and mice, gestational day (GD) 1–10, GD 10–20, and postnatal days (P) 1–10 can be considered as the equivalents of first, second, and third trimester human pregnancy, respectively [30]. Prior research on rat models exposed to moderate prenatal alcohol dosages revealed ultrastructural changes in brain capillaries at P20–30 [31]. In a study using rat models, the oral administration of 6.6 g/kg of ethanol from P4 to P10 was evaluated at P10 for any alterations in brain microvasculature [32]. There was no change in capillary density, while capillary diameters were increased in the cerebellum and hippocampus regions, unlike the dentate gyrus region [32]. Later, in a mouse model (at P2) of the third trimester equivalent of human pregnancy demonstrated the loss of radial orientation of the microvessels and a reduction in cerebral vascular density when maternal injection of 3 g/kg ethanol occurred during GD 13–19, which was a time-line equal to the second trimester of human pregnancy [33]. Overall, these rodent studies suggest that alcohol exposure during the human mid and late trimesters might affect the microvasculature differentially in the brain regions.

Human embryos are susceptible to ethanol. Evidence suggests that exposure to high amounts of ethanol during human development causes craniofacial, cardiovascular, and neurological abnormalities, which are often accompanied by cognitive and behavioral deficiencies. Autopsy and magnetic resonance imaging (MRI) investigations found that individuals who were exposed to alcohol *in utero* had structural brain damage, including lower brain sizes and reduced amounts of white and gray matter inside the brain [reviewed in 4]. However, the animal research mentioned earlier suggests that exposure to PAE during late gestation might be perilous. In a postmortem examination of human brain tissues from fetuses that deceased spontaneously *in utero*, stage-dependent alterations in the cortical vascular network were detected in the cortex of fetuses with FAS. The radial arrangement of cortical microvessels was markedly disrupted in FAS patients after 30 weeks of gestation, whereas no changes were seen in alcohol-exposed human embryonic brain tissues between 20 and 22 weeks of gestation. In addition, dynamic microscopy techniques indicated that alcohol altered endothelial cell activity and survival as well as the plasticity of the microvessel [33].

In conclusion, investigations conducted on rodents and humans suggest that prenatal alcohol consumption, especially during the late stages of gestation, may affect the macroscopical or microscopical structure of the cerebral microvascular network. These age-dependent abnormalities can be theorized because of a disturbance in cortical angiogenesis.

## Functional changes in cerebrovascular circulation due to PAE and their possible implications

Critical to brain function is a coherence among the metabolic needs, the supply of oxygen and nutrients, and the elimination of cellular waste. This matching requires continual modulation of CBF, which may be divided into four basic categories: autoregulation, vascular reactivity, neurovascular coupling (NVC), and endothelium-dependent responses. Due to the limited scope of this paper, this part of the review will discuss how PAE affects the functional parts of cerebrovascular circulation, with focuses on how it changes CBF and vascular reactivity and the clinical effects of these changes.

### Changes in CBF

The effects of maternal alcohol intake during pregnancy on outcome depends on the quantity and patterns of alcohol consumption. In animal trials, binge-like drinking patterns, in which the unborn is exposed to elevated blood alcohol concentrations (BACs) during relatively brief intervals, were shown to be more detrimental, even when the overall quantity of alcohol taken was lower than that of more continual drinking patterns [34, 35, 36]. Because binge drinking produces high BACs, may occur at important phases of brain development, and can be coupled with recurrent withdrawal episodes, it may be extremely detrimental [35]. In addition to a single binge pattern, recurrent binge-like events of maternal ethanol consumption are harmful to the fetus likewise [35] and may cause transitory and chronic abnormalities in cerebrovascular functioning. Abrupt CBF alterations in embryos may be associated with craniofacial deformity, fetal growth limitation, neuronal death, impaired delivery of nutrients to and elimination of metabolites from neurons, and a reduction in cerebral arterial tone. Here, we will examine how varied patterns of maternal alcohol use affect the blood flow to the cerebral arteries.

#### Temporary effects of maternal alcohol consumption on CBF

Following acute ethanol consumption, previous research on pregnant murine [19], ovine [41, 40, 39, 38, 37], and baboon [46, 45, 44, 43, 42] have showed aberrant uterine and cerebral blood flow. During acute maternal alcohol intake *in vivo*, the majority of investigations observed an increase in fetal cerebral perfusion and, perhaps, a decrease in fetal cerebral artery blood flow doppler velocity indices [46, 38, 42, 19]. In contrast, maternal infusions of 1 g/kg ethanol were associated with a reduction in CBF in preterm sheep [39] and baboons [45]. There was no decline in fetal CBF in mid-gestation sheep [40]. In this trial, 1 g/kg of ethanol was infused into the maternal blood at gestational day 92 (human equivalent term at 145–150 days), resulting in a maternal BAC of 150 mg/dl [40]. In contrast, in a separate experiment using the same animal model and a comparable experimental technique, Parnell et al. (2007) found that a greater dosage of ethanol (1.75 g/kg) substantially enhanced CBF by over 30 percent. Specifically, in the cerebellum, the rise in CBF 1 h after ethanol infusion was up to 50 percent greater than in the control group. These two experiments together demonstrated that, *in vivo*, the effects of alcohol on cerebral blood flow were concentration-dependent and brain region-specific.

In addition, the alterations in fetal CBF were detected along with the changes in systemic hemodynamics: a considerable increase in fetal cardiac output and heart rate, as well as a decrease in mean arterial pressure and systemic peripheral resistance [41]. An ultrasonography investigation demonstrates that acute single and recurrent binge-like episodes of maternal ethanol intake may promptly and chronically alter cranially-directed fetal blood flow throughout the second trimester [19]. PAE not only induces alterations in CBF but also impairs autoregulation. Additionally, PAE also modulates CBF responses to environmental variables. It was shown that fetal cerebral vasodilator responses to hypoxia [47] and acidosis [48] are altered by exposure to ethanol during the second trimester. Earlier work in an ovine model of pregnancy showed that PAE (1 g/kg ethanol maternal infusion i.v. for 3 weeks during the equivalent of the first trimester) reduced the adaptive rise in CBF in response to hypoxia in 1–4-day-old term lambs. As a consequence, neonatal brain oxygen supply could not be sustained [49]. Thus, it demonstrates that PAE as early as the first trimester might increase cerebral vascular susceptibility to environmental damage. Changes in the blood’s metabolic profile, such as acidemia and hypercapnia, often follow acute alcohol-induced disruption of CBF and may contribute to its clinical effects [41, 40, 39].

#### Persistent effect of maternal alcohol consumption on CBF: Impairment of vascular reactivity

Multiple groups have explored the influence of PAE on fetal cerebrovascular function. A study, utilizing doppler ultrasonography revealed that in a baboon model of pregnancy, diminution of PAE alcohol impact occurred earlier to delivery. That study particularly indicated that acute fetal alcohol intake, with a maternal BAC of 80 mg/dl in the equivalent of the second trimester of human pregnancy, decreased cerebral blood flow in fetal cerebral arteries. However, this influence on fetal vascular function did not persist throughout the duration of pregnancy [45]. This indicates that alcohol-induced changes in the physiological characteristics of the fetal cerebral arteries disappear with development. In another example of an ovine PAE model, the alcohol impact of PAE diminished after delivery. Adult cerebral artery dilatory effects to adenosine A2A receptor agonist, CGS21680, were investigated *in vitro* using arterioles extracted from third trimester comparable ovine fetuses exposed to ethanol *in utero*. The dilatory reaction to micromolar doses of CGS21680 was substantially greater when compared to control group [48]. However, when arterioles were collected from adult sheep using a similar ethanol administration approach [50], same dilator responses were comparable to the control counterpart. Therefore, experiments using diverse animal models suggested that PAE-induced abnormalities in the pharmacological nature of cerebral arteries were slowly reversed with aging.

Turcotte et al. found that prenatal exposure to ethanol (6.4%) lowered the relaxation of the aorta to carbamylcholine in rats 25 weeks after birth, indicating a change in the reactivity of peripheral arteries [51]. Similarly, in peripheral arteries, some of the alcohol-induced modifications in fetal cerebral artery characteristics may persist and be detectable long after birth. Multiple studies on rats reveal the long-lasting effects of PAE. *In vivo* responses of cerebral arterioles to eNOS- and nNOS-dependent agonists were reduced in young rats (4–6 weeks old) [52] and in adult rats (12–15 weeks old) [53,54] exposed to alcohol during fetal development. While it is known that prenatal alcohol exposure impairs the dilatation of cerebral arterioles in rats, no research has explored the effect of prenatal alcohol on the constrictor response of cerebral arterioles until recently. The response of cerebral arterioles to U-46619 (a thromboxane-mimetic analog) and arginine vasopressin (AVP), which are physiologically important constrictors, was comparable in male and female rats independent of prenatal alcohol exposure and age. Similarly, there was no difference between male and female adolescent rats’ responses to angiotensin II after prenatal alcohol consumption. At adulthood, however, alcohol-exposed females demonstrated an unanticipated dilatation in response to a high concentration of angiotensin II, but males did not. Except in adult female rats, the majority of the vasoconstriction responses to prolonged prenatal alcohol exposure were retained [55]. Besides inducing the loss of vascular reactivity, PAE is capable of inducing arterial stiffness to the cerebral vasculatures [56].

### Implications

Long-term research on humans have previously proven that offspring of binge-drinking mothers demonstrate particularly significant cognitive and behavioral abnormalities. The ability of the brain to maintain adequate cerebral blood flow in the face of changes in metabolic demand may influence the pathogenesis of symptoms associated with FASD in adults, i.e., cognitive decline, psychiatric symptoms, dementia, and seizures, all of which may be directly impacted by the ability of the brain to maintain adequate cerebral blood flow (neurovascular coupling) [57–60].

In addition, the loss of cerebrovascular autoregulation, such as during severe hypertensive encephalopathy, may result in catastrophic occurrences, such as subarachnoid hemorrhage and hemorrhagic stroke [61]. Evidently, our lab has also shown that in prenatal exposure to alcohol exacerbated brain damage in adult rats after ischemia/reperfusion and that treatment of dams with apocynin reduced this increase in brain injury following ischemia/reperfusion [53]. Thus, it may be inferred that PAE-induced alterations in cerebral blood flow not only contribute to the pathophysiology of fetal alcohol syndrome, but also have the potential to cause serious brain injury.

## Molecular and cellular mechanisms of impairment and recent preclinical interventions

Because alcohol is a simple ligand that may concurrently target several chemical entities, its effects on the developing brain are very complicated. Alcohol may cause cell death in some types of brain cells while interfering with the cellular and molecular activities of other types. These effects may be caused by alcohol both directly and indirectly.

Alcohol may have direct effects on embryonic brain development by interfering with neuronal proliferation and migration [62] or by inducing cell death [63,64,65]. In addition, alcohol may raise fetal glutamate levels [66, 67] and decrease glutamate N-methyl-D-aspartate receptors [68, 69], which may result in aberrant neuronal and glial migration.

Alcohol-induced hypoxia in the fetus is a significant indirect cause of alcohol. Alcohol reduces blood flow to the umbilical artery [70, 71], which might result in growth retardation [72, 73]. In addition to inhibiting protein synthesis and altering hormone levels, alcohol may further impede development [74, 75]. Increased oxidative stress on the embryo [76, 77] and disruption of growth factor signaling [78, 79] are additional pathways.

These effects can be toxic (short-term) and teratogenic (long-term). This section will examine as well as summarize (Table 1) the toxic and teratogenic processes that generate short-term or long-term effects on the cerebral arteries and microvessel network.

**TABLE 1:** Multiple cellular and molecular mechanisms of cerebrovascular impairment due to prenatal alcohol exposure.

Summary Table: Molecular and cellular mechanisms of cerebrovascular impairment due to prenatal alcohol exposure
❖ **Mechanism of transient effects of prenatal alcohol exposure:**
A. Cerebrovascular layer involved: Cerebrovascular myocyte (mainly)
• Through eCB system: CB receptor types 1 and 2 [46].
− Preclinical agent used for improvement: Rimonabant, a CB receptor inverse agonist [46]
❖ **Mechanism of persistent effects of prenatal alcohol exposure:**
A. Cerebrovascular layer involved: Cerebrovascular myocyte
1. Through increasing anandamide (AEA)-induced dilatation [46].
B. Cerebrovascular layer involved: Cerebrovascular endothelial cells
2. Through increased expression of collagen and tropoelastin, leading to increased stiffness of the vessel [56]
3. Through eNOS and nNOS-dependent impairment, leading to decreased reactivity of the vessel [52, 53]
4. Through increased generation of ROS [52, 53]
−Preclinical agent used for improvement:
• Apocynin (non-specific action) [52, 53].
• Rosiglitazone (specific action) [54].
5. Through decreased 5-hydroxytriptamine mediated vasodilation [56].
C. Extravascular
6. Through neurohormonal alteration
− E.g. reduces VIP levels [92, 50].
❖ **Mechanism of morphological modifications of NVC:**
a. Angiogenesis:
1. Through VEGF-mediated modification:
Preclinical agent used for improvement: Exogenous VEGF [33].
2. Through inhibition of autophagy in brain endothelial cells:
1. Enhanced expression of microtubule-associated protein (LC3) and ubiquitin-binding protein (p62) in endothelial cells [94].
2. Downregulation of the mTOR pathway.
− Preclinical agent for improvement: mTOR inhibitor, rapamycin [94].
b. Endothelial cell survival:
1. Through generation of ROS > mitophagy > cell death [96].
2. Through releasing of AIF > impaired mitochondrial integrity
> cell death.
− Preclinical agent used for improvement: Activation of autophagy by rapamycin [94].
3. Through Mitochondria-linked cellular apoptosis [97]
− Preclinical agent used for improvement: ROS scavengers and antioxidants.
❖ **Mechanism of loss of integrity of NVC:**
1. Through enhanced permeability of BBB: anticipated (no published evidence yet).
2. Through MMP-induced proteolysis of the neurovascular matrix [99,100,101].

Expansion of abbreviations used: AEA, N-arachidonoylethanolamine; AIF, apoptosis-inducing factor; BBB, blood brain barrier; CB, cannabinoid; eCB, endocannabinoid; eNOS, endothelial nitric oxide synthase; LC3, 1A/1B-light chain 3; MMP, matrix metalloproteinases; mTOR, mammalian target of rapamycin; NVC, neurovascular coupling; nNOS, neuronal nitric oxide synthase; ROS, reactive oxygen species; VEGF, vascular endothelial growth factor; VIP, vasoactive intestinal peptide.

### Mechanism of transient and persistent effects of prenatal alcohol exposure

Alcohol’s short-term effects on CBF are highlighted by ethanol’s targeting of molecular players inside the fetal cerebral blood vessel. In particular, PAE is characterized by fetal cerebral artery dilatation in the presence of alcohol, which is mediated by cannabinoid (CB) receptors 1 and 2. A study showed that the endocannabinoid (eCB) system is a target of maternal alcohol intake inside the fetal cerebral arteries, and that rimonabant, a CB receptor inverse agonist, might be a potential rescue treatment, as suggested by Seleverstov et al. [46]. Furthermore, fetal CB2 receptor-mediated cerebral artery dilation by anandamide was up-regulated in alcohol-exposed fetuses, showing that these receptors are the primary determinants of the persistent vasodilatory impact of alcohol on fetal cerebral arteries [46]. In addition to the increase of anandamide (AEA)-induced dilatation, other mechanisms of PAE’s lasting effects are described in subsections below.

#### Endothelial nitric oxide synthase

Endothelial nitric oxide synthase (eNOS) is extremely susceptible to prenatal ethanol exposure in fetal cerebral arteries. In fetal cerebral arteries of an ovine model of pregnancy - where fetal plasma alcohol concentration reached 108 mg/dl during the late gestational period of day 95–133 (human equivalent term pregnancy) - a decreased endothelium-dependent vasodilation was observed in response to the dilator 5-hydroxytriptamine in alcohol-exposed donors. Authors also discovered a substantial reduction in endothelial nitric oxide synthase (eNOS) mRNA [56]. In contrast, in adolescent rats (4–6 weeks old) and adult rats (12–15 weeks old), *in utero* alcohol exposure decreased cerebral arteriole responses to eNOS (ADP) and neuronal nitric oxide synthase (nNOS)-dependent (NMDA) agonists [52, 53]. However, in microvessels and tissue from the parietal cortex of adolescent rats, the expression of eNOS and nNOS levels was not altered [52].

#### Stiffness of the arterioles

In the ovine model mentioned above, Parkington et al. also discovered that PAE significantly increased the fetal cerebral artery’s total functional stiffness. Evidently, the elastic modulus of arteries in alcohol-exposed groups was approximately double that of control groups. In comparison to the control group, the alcohol-exposed group showed considerably higher mRNA levels for collagen Ia1 and tropoelastin, revealing the mechanism behind the enhanced functional stiffness of the vessel [56].

#### Increasing production of Reactive Oxygen Species (ROS)

**FIGURE 1:**
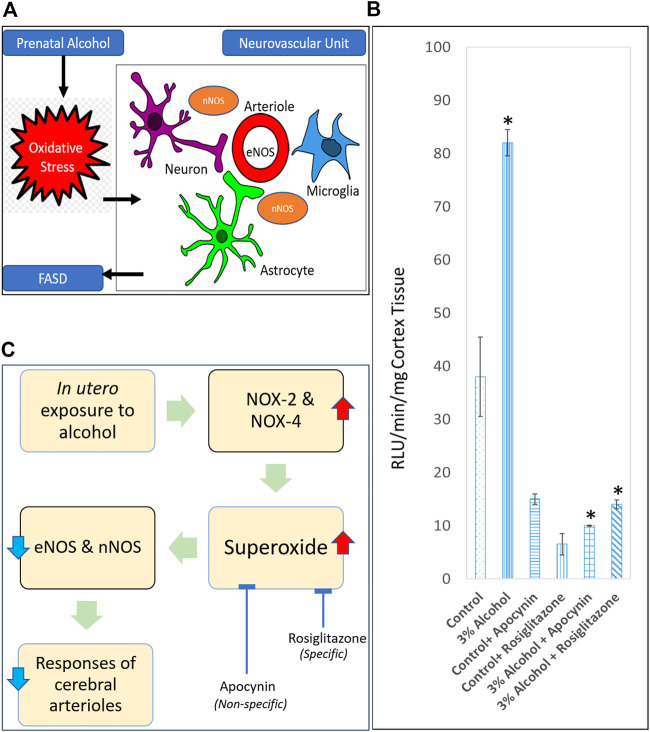
Role of oxidative stress in causing neurovascular damage in FASD. **(A)** Endothelial nitric oxide synthase (eNOS) and neuronal nitric oxide synthase (nNOS) are some of the targets for prenatal alcohol-induced oxidative stress in the neurovascular unit. **(B)** Superoxide levels at baseline in parietal cortex tissue from adult control rats, rats exposed to alcohol *in utero*, control + apocynin rats, control + rosiglitazone rats, alcohol + apocynin, and alcohol + rosiglitazone rats [Adapted and combined from 53 & 54 with proper permission from publisher, John Wiley and Sons]. *p < 0.05 *versus* levels before treatment with apocynin and rosiglitazone. **(C)** Proposed pathway for a physiological relationship between *in utero* alcohol exposure and impaired cerebral arterioles due to superoxide production. Expansion for NOX = NADPH oxidase.

EtOH and its catabolite acetaldehyde are itself harmful, although oxidative stress is the predominant damaging mechanism, according to current understanding. Oxidative stress is characterized by high intracellular levels of reactive oxygen species (ROS) that cause lipid, protein, and DNA damage. ROS are molecules or ions generated by the incomplete reduction of oxygen by one electron. The principal ROS species are superoxide (O2•), hydrogen peroxide (H2O2), and the hydroxyl radical (•OH). Cananzi and Mayhan evaluated the production of superoxide in the cerebral arterioles of prenatally alcohol-exposed adolescent and adult rats [52, 53], given that oxidative stress has been found to enhance neurovascular and neuronal damage and death in multiple brain locations in FASD (Figure 1A). PAE increased the baseline levels of superoxide in the parietal cortex tissue of rats (Figure 1B), even when NADPH, which was used to enhance NADPH oxidase activity, was administered. NOX-2 and NOX-4 are isoforms of the NADPH oxidase system that are found in endothelium and vascular smooth muscle and play a crucial role in the generation of superoxide [80–84]. Alcohol exposure during gestation increases the expression of NOX-2 and NOX-4 in microvessels. In addition, they discovered that apocynin, a powerful antioxidant, lowered superoxide levels (Figure 1C) and relieved impairment of eNOS- and nNOS-dependent responsiveness of cerebral arterioles in rats exposed to alcohol during gestation. This indicated an increase in superoxide generation in these animals, which may lead to an increase in oxidative stress in rats exposed to alcohol during gestation [52, 53]. Importantly, they discovered that apocynin reduces the likelihood of brain injury in adult rats after cerebral ischemia [53]. As apocynin was a nonspecific inhibitor of superoxide anion, the particular subcellular route underlying the impairment of cerebral vascular function remained unclear. Saha et al. used rosiglitazone, an agonist for the gamma subtype of peroxisome proliferator-activated receptors (PPARs) to determine the specific vascular anti-oxidant mechanism [54]. It has been shown that these receptors sit on vascular smooth muscle and endothelium [85,86,87]. In addition, both acute and chronic treatment of male and female adult rats with rosiglitazone protected decreased eNOS and nNOS-dependent vascular function in alcohol-exposed male and female adult rats. In addition, acute rosiglitazone decreased superoxide levels (Figure 1C) in parietal cortex tissue, indicating the anti-oxidant mechanism(s) *via* which rosiglitazone enhanced vascular function [54]. PAE (three percent ethanol for entire gestation period) induced dysfunction in the ability of specific potassium channels to dilate in rat cerebral arterioles. This dysfunction appears to be mediated by an increase in oxidative stress, as acute apocynin was able to enhance the response [unpublished data].

#### Neurohormonal alteration

Vasoactive intestinal peptide (VIP) operates as a nonadrenergic, noncholinergic neurotransmitter or neuromodulator in both the peripheral and central nervous systems, where it acts on specific receptors to dilate cerebral arteries, pial arterioles, and intracerebral arterioles [88–91]. Alcohol exposure during gestation decreases VIP levels permanently in the rat fetal brain [92]. Alcohol exposure during pregnancy dramatically affected the dilator response of adult intracerebral arterioles to VIP in an adult sheep model of binge-drinking during pregnancy [50]. This decrease may suggest a loss of neuronal connections, including neurons carrying VIP, or a change in VIP receptor density in the adult brain. This is the first research to indicate that exposure to alcohol during fetal development may have long-lasting consequences on vasomotor responses in adult brain arteries.

### Mechanism of modifications of morphology and integrity of NVC in response to prenatal alcohol exposure

PAE has profound impacts on angiogenesis and endothelial cell survival on the formation of cortical blood vessels. The molecular processes behind these effects are poorly understood. VEGF is an effective blood vessel development regulator. VEGF-R1, VEGF-R2, and VEGF-R3 receptors [93] mediate the biological actions of VEGFs. Jégou et al. [33] evaluated VEGF-R1 and VEGF-R2 protein levels in cerebral microvessel extracts of P2 mice exposed to prenatal alcohol in 2012. They observed an upregulation and also a simultaneous downregulation of VEGF1 and VEGF2 receptor proteins respectively in the cortical microvascular network during PAE; these changes were found to be associated with detrimental alterations in density and radial organization. Thus, disruption of these receptor subtypes is one of the mechanisms behind the VEGF-mediated modification of the cerebral microvascular network in rats prenatally exposed to alcohol. One of their *in vitro* experiments revealed that exogenous VEGF reduced the deleterious effect of ethanol on the vascular plasticity of the cortical glia [94].

During fetal development, well-controlled autophagy promotes vascular development and also protects against autophagic cell death. It is especially vital in ECs, one of the principal components of the developing blood vessels, as they help to adjust their bioenergetic and biosynthetic requirements in response to shifting environmental conditions, the presence of angiogenic stimuli, or intrinsic and extrinsic damages. PAE may inhibit autophagy in brain endothelial cells, hence leading to changes in angiogenesis and the resultant brain abnormalities identified in individuals with pFAS/FAS. PAE increases the frequency of autophagic vacuoles in the endothelium of cortical microvessels in human fetal brain tissues and in a mouse model of PAE in neonates, indicating defective autophagy [94, 95]. Girault et al. discovered that the levels of autophagy marker proteins, such as microtubule-associated protein 1A/1B-light chain 3 (LC3) LC3 and ubiquitin-binding protein p62, were considerably enhanced in endothelial cells treated with 50 mM ethanol in order to understand the process. In addition, a reduction in Rab7, a protein that plays a crucial function in endocytosis, was detected, which may account for the impaired autophagosome–lysosome fusion. Importantly, these effects of ethanol were eliminated in the presence of 4-methylpyrazole, which inhibits the synthesis of the ethanol metabolite acetaldehyde. These findings showed that acetaldehyde (MeCHO) triggers the process of autophagy dysregulation in cerebral microvessels after alcohol exposure [94].

In addition, it was shown that the increase in autophagy vacuoles after alcohol exposure was associated with a downregulation of the mammalian target of rapamycin (mTOR) pathway. They also demonstrated that activating autophagy with the mTOR inhibitor rapamycin reduces ethanol-induced endothelial cell death and restores vascular plasticity [94]. Overall, this shows that ethanol adversely affects angiogenesis by promoting endothelial autophagy in the cortical layer.

Previous research has shown that the neuroprotection afforded by autophagy may originate from the elimination of damaged mitochondria. And the lowered degree of autophagy may promote ROS production and excessive mitophagy [96], hence enhancing ethanol-induced cell death. In murine pulmonary microvascular endothelial cells (MPMVEC), Girault et al. found that ethanol impairs mitochondrial integrity and triggers apoptosis-inducing factor (AIF) protein release and nuclear translocation, which may result in programmed cell death. Therefore, they hypothesized that activation of autophagy by rapamycin may similarly shield endothelial cells from ethanol-induced mortality and aid cortical angiogenesis in individuals with pFAS/FAS [94].

Among numerous pathways of cell death generated by fetal brain alcohol exposure, fetal cerebral artery mitochondria-linked cellular apoptosis has been documented in an animal model of prenatal alcohol exposure and FASD-related brain injury [97]. Alcohol-induced abnormalities in prenatal cerebrovascular mitochondria may result from both direct targeting by alcohol [29, 98] and secondary damage resulting from alcohol-induced changes in fetal cerebral blood flow [41, 46]. Therefore, mitochondria-targeted therapies with ROS scavengers and antioxidants might be a viable therapeutic strategy for the treatment of FAS/FASDs.

The integrity of cortical microvessels is necessary for optimal vascular development. Permeability of the BBB and matrix metalloproteinases (MMP)-induced proteolysis of the neurovascular matrix may influence cortical vascular development. BBB permeability has also been proposed as a possible site of PAE-induced change in cerebral capillaries. However, no documented evidence of prenatal ethanol-induced BBB permeability currently exists. In contrast, MMP-induced proteolysis of the neurovascular matrix may also cause programmed cell death by cell separation from the extracellular matrix [99, 100]. Indeed, glutamate-induced activation of the endothelium protease MMP-9 from pial microvessels of neonates was seen in a mouse model of PAE [101].

As yet, no global mechanism of alcohol-induced impairment to embryonic or fetal brain development has been revealed, and it is very unlikely that a single mechanism can explain the various components of the FASD presentation. In addition, while alcohol is often regarded the principal chemical that causes birth defects (i.e., a teratogen), alcohol’s breakdown products (i.e., its metabolism) may also play a role. For instance, acetaldehyde, a toxin produced by the breakdown of alcohol in the liver and other organs, may accumulate in the fetal brain during prenatal alcohol exposure and may contribute to the development of FASD. Each individual shows a unique mix of alcohol-related consequences, which is influenced by the time, amount, pattern, and length of the mother’s drinking, in addition to hereditary variables. This variation makes it difficult to compare the effects of drinking across individuals.

## Conclusion

There is currently no readily available treatment for intentional or unintentional alcohol intake during pregnancy. The inadequate mechanistic knowledge of FASD’s pathogenesis is one of the explanations. More research is required to understand the underlying mechanisms through which alcohol might affect the structure and function of cells. Understanding these multiple mechanisms and seeking to inhibit them may help us to reduce the negative effects of alcohol exposure on embryonic development in the future. Additionally, efforts must be made to improve public awareness of the detrimental consequences of even little alcohol intake during pregnancy.
